# Changes in 24-hour blood pressure profile after 12 weeks of dapagliflozin treatment in patients with diabetic kidney disease: an Italian multicenter prospective study

**DOI:** 10.1093/ckj/sfae316

**Published:** 2024-10-17

**Authors:** Silvio Borrelli, Carlo Garofalo, Gianpaolo Reboldi, Annapaola Coppola, Paolo Chiodini, Mariadelina Simeoni, Alessio Mazzieri, Luca Della Volpe, Maurizio Gallieni, Carola Zummo, Santina Cottone, Maura Ravera, Filippo Aucella, Francesco Aucella, Giovanni Stallone, Valeria Gismondi, Federico Alberici, Marco Gregori, Giuseppe Castellano, Simone Vettoretti, Mario Cozzolino, Chiara Ruotolo, Roberto Minutolo, Luca De Nicola

**Affiliations:** Unit of Nephrology, Dept of Advanced Medical and Surgery Sciences, University of Campania “Luigi Vanvitelli”, Naples, Italy; Unit of Nephrology, Dept of Advanced Medical and Surgery Sciences, University of Campania “Luigi Vanvitelli”, Naples, Italy; Department of Medicine, University of Perugia, Perugia, Italy; Unit of Nephrology, Dept of Advanced Medical and Surgery Sciences, University of Campania “Luigi Vanvitelli”, Naples, Italy; Medical Statistics Unit, University of Campania “Luigi Vanvitelli ” , Naples, IT, Italy; Department of Translational Medical Sciences, University of Campania Luigi Vanvitelli, Naples, Italy; Specialty School of Nephrology, University of Milano, Milan, Italy; Specialty School of Nephrology, University of Milano, Milan, Italy; Specialty School of Nephrology, University of Milano, Milan, Italy; Department of Biomedical and Clinical Sciences, University of Milano, Milan, Italy; AOUP “Paolo Giaccone” Palermo, Italy; AOUP “Paolo Giaccone” Palermo, Italy; Nephrology, Dialysis and Transplantation Unit, Policlinico San Martino, Genoa, Italy; Nephrology and Dialysis Unit, Research Hospital “Fondazione Casa Sollievo della Sofferenza”, San Giovanni Rotonda (FG), Italy; Nephrology and Dialysis Unit, Research Hospital “Fondazione Casa Sollievo della Sofferenza”, San Giovanni Rotonda (FG), Italy; Nephrology, Dialysis and Transplantation Unit, Department of Medical and Surgical Science, University of Foggia, Foggia, Italy; Nephrology, Dialysis and Transplantation Unit, Department of Medical and Surgical Science, University of Foggia, Foggia, Italy; Nephrology Unit, University of Brescia, ASST Spedali Civili, Brescia, Italy; Nephrology Unit, University of Brescia, ASST Spedali Civili, Brescia, Italy; Fondazione IRCCS Ca’ Grande Ospedale Maggiore Policlinico di Milano, Milan, Italy; Fondazione IRCCS Ca’ Grande Ospedale Maggiore Policlinico di Milano, Milan, Italy; Renal Division, Department of Health Sciences, University of Milan, Milan, Italy; Unit of Nephrology, Dept of Advanced Medical and Surgery Sciences, University of Campania “Luigi Vanvitelli”, Naples, Italy; Unit of Nephrology, Dept of Advanced Medical and Surgery Sciences, University of Campania “Luigi Vanvitelli”, Naples, Italy; Unit of Nephrology, Dept of Advanced Medical and Surgery Sciences, University of Campania “Luigi Vanvitelli”, Naples, Italy

**Keywords:** ambulatory blood pressure, diabetes kidney disease, nocturnal hypertension, SGLT2 inhibitors

## Abstract

**Background:**

Sodium-glucose cotransporter 2 inhibitors (SGLT2i) lower ambulatory blood pressure (ABP) in patients with type 2 diabetes mellitus; whether the same holds true in diabetic kidney disease (DKD) is unknown. This information is critical to the knowledge of mechanisms of nephroprotection and safety of this therapy.

**Methods:**

This multicenter prospective study evaluates the changes in ABP after 12 weeks of dapagliflozin 10 mg/day in a cohort of patients with type 2 DKD and glomerular filtration rate (GFR) >25 mL/min/1.73 m^2^. Primary endpoint was the change of nighttime systolic blood pressure (SBP). Changes of daytime SBP, prevalence of normal dipping (day/night SBP ratio <0.9) and changes in ABP patterns, that is, sustained uncontrolled hypertension (SUCH), white coat uncontrolled hypertension (WUCH), masked uncontrolled hypertension (MUCH) and controlled hypertension (CH) were secondary endpoints.

**Results:**

Eighty-three of 96 patients completed the study [age 68.7 ± 8.9 years, 73.5% males, GFR 49 ± 17 mL/min/1.73 m^2^, median albuminuria: 0.18 (interquartile range 0.10–0.38) g/24 h]. After 12 weeks of dapagliflozin, nighttime SBP declined by −3.0 mmHg (95% confidence interval −5.2/−0.8 mmHg; *P* = .010) with an improvement of nighttime SBP goal (<110 mmHg) from 18.0% to 27.0% (*P* < .001). Similarly, the prevalence of normal dipping increased (from 31.3% to 50.6%, *P* = .005). A decrease in daytime (−2.4 mmHg; *P* = .046) and office (−7.9 mmHg; *P* = .009) SBP was also found. The decline of ambulatory and office SBP was associated with increased prevalence of CH (from 6.0% to 18.0%) and significant improvement of SUCH, WUCH and MUCH (*P* = .009). Albuminuria decreased (*P* < .001), whereas eGFR did not change (*P* = .297). Urinary tract infection (4.2%) and acute kidney injury (3.6%) were the main causes of drop-out. Only one patient showed a drop of nighttime SBP below 90 mmHg.

**Conclusions:**

Dapagliflozin is associated with improvement in circadian blood pressure rhythm with no major safety signal related to excessive blood pressure decrease.

KEY LEARNING POINTS **What was known:**

- Treatment of patients with diabetic kidney disease (DKD) with dapagliflozin is associated with a significant improvement of cardiorenal outcome.
- Ambulatory blood pressure (ABP) monitoring has a greater prognostic value versus comparison with office blood pressure (BP) in patients with renal disease.
- The effect of dapagliflozin on 24 h BP in patients with DKD is unknown.

**This study adds:**

- In a cohort of patients with DKD followed in outpatient renal clinics, 12 weeks of dapagliflozin treatment was associated with a significant reduction of nighttime systolic BP and a subsequent improvement in nocturnal hypertension and dipping.
- Dapagliflozin contributed to the normalization of ABP profile, without causing excessive BP drop.

**Potential impact:**

- Decrease in nighttime systolic BP and, in general, improvement of 24 h BP profile may act as additional mechanism of cardiorenal protection in DKD patients treated with dapagliflozin.

## INTRODUCTION

The global impact of diabetes is dramatic, with over 500 million individuals affected worldwide [1]. Even more worrisome is the estimate that almost half of patients with diabetes develop diabetic kidney disease (DKD) which, in turn, acts as the leading cause of end-stage kidney disease (ESKD) [2]. Sodium-glucose cotransporter 2 inhibitors (SGLT2i) have significantly changed the treatment landscape of DKD as they are highly efficacious in preventing ESKD and cardiovascular events [3–5].

The cornerstone of the therapeutic approach to DKD is the optimal control of blood pressure (BP). In this picture, it is relevant that SGLT2i have shown an antihypertensive effect over the 24 h, similar to low-dose thiazides, in patients with diabetes without CKD [6, 7]; however, whether this finding extends to the DKD population remains unexplored to date. Indeed, ambulatory blood pressure (ABP) monitoring is the gold standard for evaluating the true BP burden in CKD, with abnormal BP patterns being more strongly linked than high office BP to poor renal and cardiovascular outcome [8–10]. In particular, elevated nighttime systolic BP (SBP) is the major component associated with risk of progression to ESKD and onset of fatal and nonfatal cardiovascular events in patients with CKD followed in tertiary nephrology care [8–11]. In fact, guidelines now consider ABP as essential tool to gain proper risk stratification in patients with CKD with and without diabetes [12, 13].

ABP, moreover, can add important information on the safety of antihypertensive drugs. In this regard, it is important to evaluate whether SGLT2i determine an excessive drop of out-of-office BP, which may increase the risk of acute kidney injury (AKI) in DKD patients who are characterized by impaired renal autoregulation [14, 15]. Indeed, despite the fact that SGLT2i have demonstrated a protective effect against AKI [5], it is also well known that patients seen in real-world nephrology practice can differ markedly from those enrolled in the trials [16].

Therefore, to fill the important knowledge gap on the 24-h BP effects in DKD, we designed a multicenter prospective study to evaluate the changes of ABP associated with the initial 12 weeks of dapagliflozin in DKD patients regularly followed in Italian outpatient renal clinics.

## MATERIALS AND METHODS

This multicenter prospective study evaluates the effects of the first 12 weeks of dapagliflozin 10 mg/day on 24 h ABP in a cohort of patients with DKD followed by 11 Italian Nephrology units.

Inclusion criteria were type 2 diabetes mellitus and estimated glomerular filtration rate (eGFR) between 60 and 25 mL/min/1.73 m^2^ or eGFR ≥60 mL/min/1.73 m^2^ and albuminuria (>30 mg/24 h). The eGFR was evaluated twice in 3 months to confirm chronic kidney failure and to exclude AKI (eGFR change >30% in the previous 3 months). Other exclusion criteria were: previous SGLT2i therapy, immunosuppressive therapy, severe cirrhosis, heart failure New York Heart Association III–IV, atrial fibrillation or inadequate ambulatory BP monitoring (daytime and nighttime readings <20 and <7, respectively) [13].

Enrolled patients gave their written informed consent. The study was conducted in accordance with the guidelines of the Declaration of Helsinki. The study protocol was approved by the Institutional Review Board (IRB no. 0 013 983 of 5 May 2022; Ethical Committee of University of Campania “Luigi Vanvitelli,” Naples, Italy) and shared with the Ethical Board of each participating center.

### Study design and procedures

Patients with DKD consecutively seen in the participating renal clinics from January 2023 to June 2023 received dapagliflozin 10 mg/day. At that time, dapagliflozin was the only gliflozin that could be prescribed in Italy by nephrologists in DKD regardless the background antidiabetic therapy. Patients were treated according to the best clinical practice; investigators were asked not to change the therapy, if possible from the clinical point of view, during the 3 months of observation.

Clinical and lab parameters and ABP monitoring (Spacelabs 90 207 devices) were obtained at baseline visit (within 1 week before starting dapagliflozin) and after 12 ± 2 weeks of treatment with dapagliflozin. ABP monitoring was performed on working days and the cuff size was chosen based on the non-dominant arm circumference. The device was set to measure BP every 15 min during the day (from 7:00 AM to 11:00 PM) and every 30 min during the night (from 11:00 PM to 7:00 AM). Daytime and nighttime were defined according to the patients’ diaries. According to the American Heart Association (AHA) hypertension guidelines, systolic ABPs were at goal if daytime SBP <130 mmHg and nighttime SBP <110 mmHg, while dipping status was considered normal if nighttime to daytime SBP ratio was <0.9 [13].

Office BP was measured during a morning visit (8:00–11:00 AM) in a sitting position thrice at 5-min intervals by the same nephrologist, who was not aware of results of ABP. Office BP value used for analyses was calculated as the mean of the six values recorded in the two consecutive days in which the Spacelabs 90 207 device was installed and removed. Office BP was used to define BP patterns as follows: sustained uncontrolled hypertension (SUCH: office SBP ≥130 mmHg, daytime SBP ≥130 mmHg or nighttime SBP ≥110 mmHg), masked uncontrolled hypertension (MUCH: office SB <130 mmHg, daytime ≥130 mmHg or nighttime SBP ≥110 mmHg), white coat uncontrolled hypertension (WUCH: office SBP≥130 mmHg; daytime SBP <130 mmHg and nighttime SBP <110 mmHg) and controlled hypertension (CH: office SBP <125 mmHg, daytime SBP <130 mmHg and nighttime BP <110 mmHg) [13].

Twenty-four-hour urine collection was performed to measure urinary excretion of albumin, creatinine and sodium. The accuracy of 24-h urine collection was assessed by urinary creatinine excretion rate (60%–140%). The eGFR was estimated using the 2009 CKD Epidemiology Collaboration equation based on serum creatinine [17]. Adverse drug reactions were collected during the 3 months of follow-up.

### Endpoint and sample size

The primary endpoint was the change in nighttime SBP from baseline to12 weeks of dapagliflozin. As secondary endpoints, we evaluated the changes from baseline in achievement of nighttime and daytime SBP goals, prevalence of normal dipping and changes of BP pattern.

Sample size was estimated based on the expected effect on the change of nighttime SBP reported in a previous meta-analysis investigating the effect of SGLT2i in diabetic patients [6]: accordingly, a sample size of 90 patients was needed to assess a mean reduction from baseline in nighttime SBP of 3 ± 10 mmHg. Sample size was calculated by STATA 14.2.

### Statistical analysis

Data for continuous variables are presented as mean and standard deviation or median and interquartile range (IQR) according to their distribution assessed with Shapiro–Wilk test. To assess differences before and after treatment, either paired *t*-test or Wilcoxon signed rank sum test were used for normally or non-normally distributed variables, respectively.

Categorical variables were presented as percentages and compared by using the Chi-square test for unpaired data, the McNemar test for paired variables and Cochran Q test for multiple comparisons.

Multiple linear regression model was used to evaluate changes from baseline in nighttime SBP. The change of nighttime SBP (dependent variable) was tested in different categories: age ≥65 or <65 years, gender, use of renin–angiotensin–aldosterone system inhibitors (RAASi), use of diuretics (thiazides or loop diuretics), history of cardiovascular disease (CVD), body mass index (BMI) ≥30 or <30 kg/m^2^; eGFR categories (≥60 or <60 mL/min/1.73 m^2^) and albuminuria categories (≥300 or <300 mg/day). Each regression analysis was adjusted for the basal nighttime SBP.

A statistical interaction was tested for each of variables included in the models. Data were analyzed using Stata 14.2.

## RESULTS

**Figure 1:**
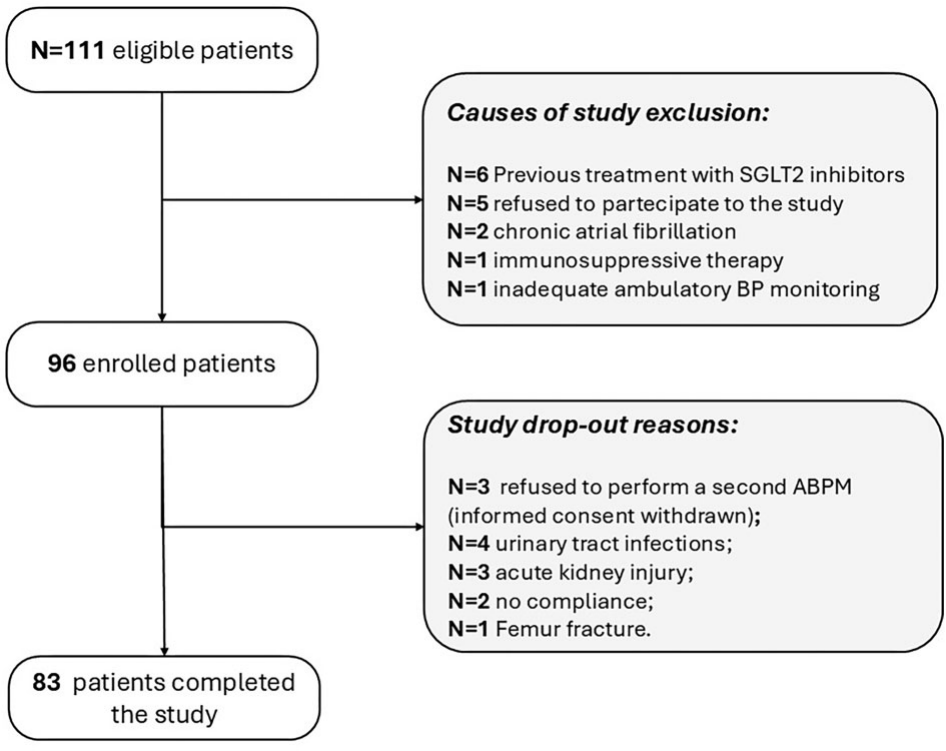
Flow chart of the study.

Out of the 111 eligible patients, 96 began the study (safety cohort) and 83 completed it (efficacy cohort) (Fig. 1). The main features of safety cohort (*N* = 96) are listed in Table 1. Thirteen patients dropped out, 10 for onset of adverse drug reactions and 3 because they refused to perform the second ABPM after 12 weeks of dapagliflozin. Causes of drug withdrawal were recurrent urinary tract infections (*n* = 4; 4.2%), AKI (*n* = 3; 3.1%), poor adherence to therapy (*n* = 2; 3.1%) and femur fracture (*n* = 1).

**Table 1:** Main clinical, lab and therapeutic features in safety cohort (*n* = 96).

Demographics	
Age (years)	68.7 ± 8.8
Male gender (%)	72.9
BMI (kg/m^2^)	28.9 ± 5.7
Prior CVD (%)	35.4
Left ventricular hypertrophy (%)	59.3
Lab	
eGFR (mL/min/1.73 m^2^)	49 ± 17
Albuminuria (mg/24 h)	180 (100–380)
Total cholesterol (mg/dL)	142 (125–174)
LDL cholesterol (mg/dL)	73(50–101)
Triglycerides (mg/dL)	127(88–173)
Hemoglobin (g/dL)	13.2 ± 1.8
Albumin (g/dL)	4.2 ± 0.4
Urinary sodium excretion (mmol/day)	
Therapy	
Number antihypertensive drugs (*n*)	2.6 ± 1.2
ACEIs or ARBs (%)	79.2
MRAs (%)	5.2
Calcium antagonists (%)	56.3
Beta blockers (%)	57.3
Alpha blockers (%)	16.7
Diuretics	
Loop (%)	27.1
Thiazides (%)	17.7
Other anti-hypertensive drugs (%)	0.0
Statin (%)	50.0
Antiaggregant (%)	49.4
Insulin treatment (%)	24.0
Metformin (%)	50.6
Other antidiabetics (%)	34.4

Data are mean ± standard deviation, median (IQR) or percentages (%).

LDL, low-density lipoprotein; ACEIs: angiotensin-converting enzyme inhibitors; ARBs: angiotensin II receptor blockers; MRAs: mineralocorticoid receptor antagonists.

The efficacy cohort (*n* = 83) was characterized by older age (>65 years in 68.7%); the majority were men (73.5%). A BMI >30 kg/m^2^ was present in 31.3% of the cohort, and 39.8% of the patients had CVD. An albuminuria ≥300 mg/24 h was recorded in 37.4%, and eGFR was <60 mL/min/1.73 m^2^ in 78.3%. Patients were treated with a mean of 2.5 ± 1.2 antihypertensive drugs with a high percentage of RAASi (80.7%). Insulin was prescribed in 26.5% of patients, metformin in 50.6%, while 34.9% received other oral anti-hyperglycemic drugs.

After 12 weeks of dapagliflozin, body weight and albuminuria decreased while hemoglobin increased (Table 2). No modification in therapy was reported in any patient throughout the 12 weeks of follow-up.

**Table 2:** Changes in main clinical, lab and therapeutic features after the first 12 weeks of dapagliflozin in the efficacy cohort (*n* = 83).

	Baseline	12 weeks	*P*
Body weight (kg)	82.8 ± 17.3	80.6 ± 16.9	<.001
BMI (kg/m^2^)	28.1 (25.2–31.7)	27.4 (24.6–31.0)	<.001
eGFR (mL/min/1.73 m^2^)	49.1 ± 16.5	50.1 ± 19.6	.297
Albuminuria (mg/day)	180 (100–380)	120 (50–310)	<.001
Potassium (mmol/L)	4.6 ± 0.5	4.5 ± 0.5	.585
Total cholesterol (mg/dL)	142 (119–173)	141 (116–160)	.168
LDL cholesterol (mg/dL)	72 (53–97)	74 (52–93)	.708
Triglycerides (mg/dL)	127 (85–170)	107 (90–153)	.283
Hemoglobin (g/dL)	13.1 ± 1.8	13.9 ± 1.7	<.001
Albumin (g/dL)	4.2 ± 0.4	4.2 ± 0.4	.967
Urinary sodium excretion (mmol/day)	125 (77–128)	113(77–168)	.512

Data are mean ± standard deviation or median (IQR).

### Ambulatory and office BP after 12 weeks of dapagliflozin

As for the primary endpoint, significant reduction in nighttime SBP was found after 12 weeks of dapagliflozin (−3.0 mmHg, 95% confidence interval −5.2/−0.8 mmHg; *P* = .010).

**Figure 2:**
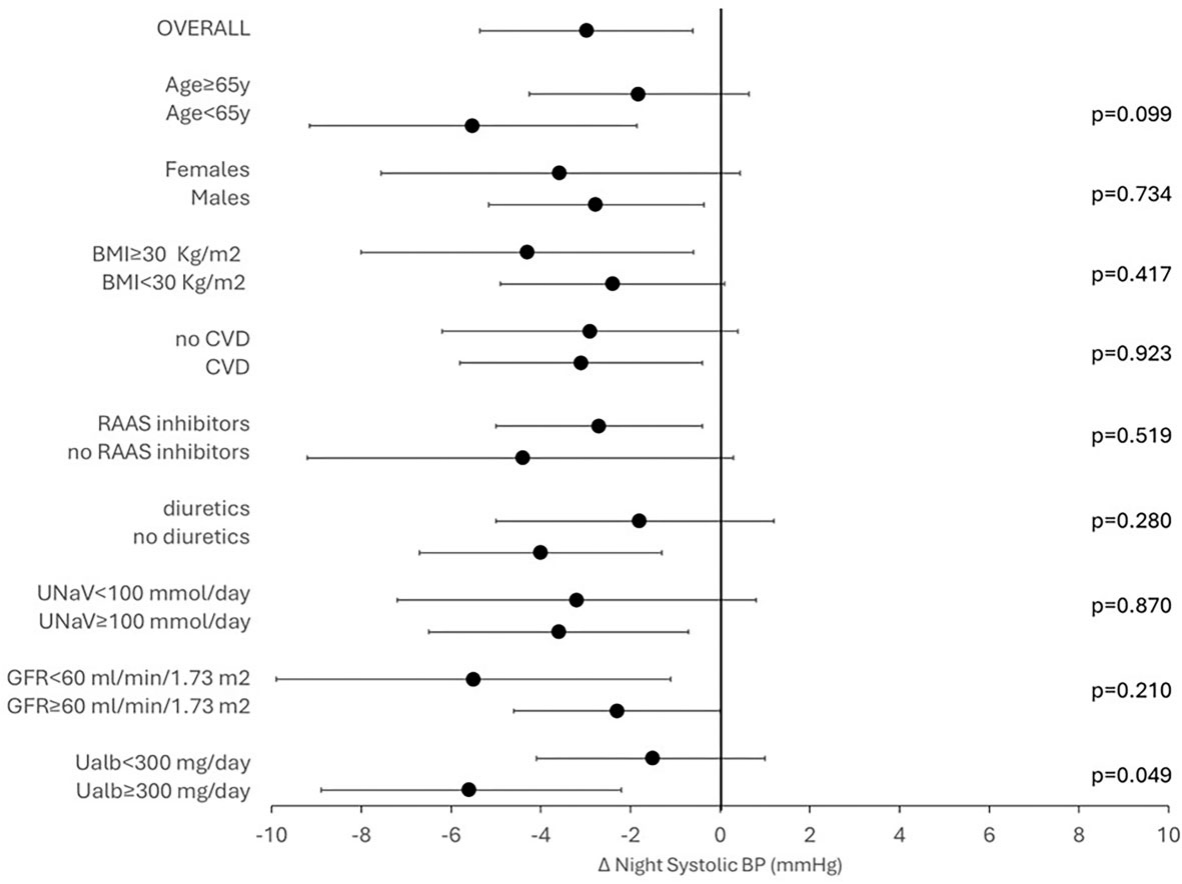
Change in nocturnal nighttime SBP after 12 weeks of dapagliflozin in whole cohort and stratified by age groups (<65 vs >65 years), gender (males vs females), obesity (BMI <30 vs $\ge$30 kg/m^2^), history of CVD, RAASi therapy (yes vs no), diuretics use, low sodium intake (<100 vs ≥100 mmol/L), eGFR (<60 vs ≥60 mL/min/1.73 m^2^) and albuminuria (30–300 vs $\ge$300 mg/day). The model was adjusted for nighttime SBP at basal visit.

As depicted in Fig. 2, the adjusted change in nighttime SBP did not differ according with age (*P* = .099), sex (*P* = .734), BMI (*P* = .417), CVD (*P* = .923), or treatment with either RAASi (*P* = .519) or diuretics (*P* = .280). Notably, a larger reduction in nighttime SBP was detected in patients with albuminuria ≥300 mg/24 h (*P* = .049), whereas no significant difference was found between eGFR strata (*P* = .210).

As reported in Table 3, a significant reduction in daytime SBP was also observed. Additionally, there was a notable decrease in office SBP (−7.9 mmHg; *P* = .009).

**Table 3:** Changes in office BP and ABP at baseline and after 12-week dapagliflozin therapy (*n* = 83).

	Baseline (mean ± SD)	Week 12 (mean ± SD)	Absolute change (95% CI)	*P*
Office SBP (mmHg)	139.5 ± 17.5	131.7 ± 12.9	−7.9 (−12.1/−3.6)	<.001
Office DBP (mmHg)	80.7 ± 13.6	76.2 ± 7.5	−4.4 (−7.6/−1.3)	.006
24-h SBP (mmHg)	128.7 ± 12.4	126.4 ± 11.0	−2.3 (−4.5/−0.2)	.032
24-h DBP (mmHg)	73.2 ± 9.1	72.1 ± 7.5	−1.1 (−2.7/0.5)	.169
Daytime SBP (mmHg)	131.6 ± 13.2	129.1 ± 11.0	−2.4 (−4.8/−0.1)	.046
Daytime DBP (mmHg)	75.7 ± 9.0	74.0 ± 7.9	−1.7 (−3.3/−0.1)	.046
Nighttime SBP (mmHg)	123.1 ± 13.6	120.1 ± 13.3	−3.0 (−5.2/−0.8)	.010
Nighttime DBP (mmHg)	67.2 ± 11.1	66.1 ± 8.4	−1.1 (−2.9/0.6)	.197
Dipping status (%)	31.3	50.6	+19.3	.005

SD, standard deviation; DBP, diastolic blood pressure; CI, confidence interval.

**Figure 3:**
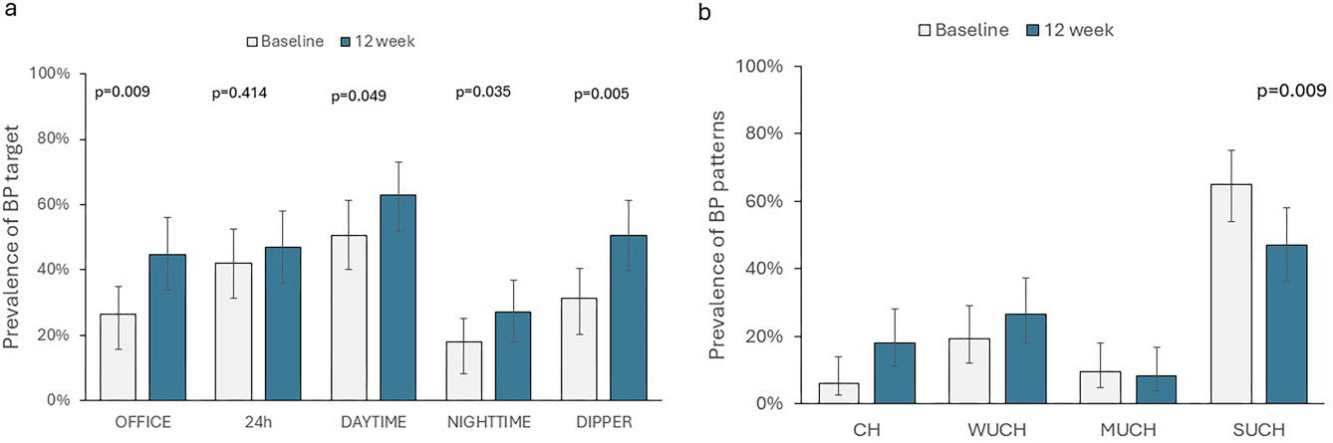
Changes in prevalence of SBP goals (3a) and BP patterns (Figure 3b) after 12 weeks of dapagliflozin.

As depicted in Fig. 3a, the achievement of nighttime SBP goal improved from 18.1.0% to 26.5% (*P* = .035. A significant improvement in SBP goal was also detected for daytime and office BP. These changes translated into a significant increase in the prevalence of CH from 6.0% to 18.1% and a decrease in the prevalence of altered BP profile (Fig. 3b). Concurrently, the ratio of nighttime/daytime SBP declined from 0.93 (IQR 0.88–0.98) to 0.90 (IQR 0.89–0.97) with a consequent improvement in the prevalence of normal dipping status (from 31.3% to 50.6%; *P* = 0.005).

## DISCUSSION

This study provides first-time analysis on the change of ABP profile associated with the first 12 weeks of dapagliflozin treatment in patients with DKD under nephrology care. We detected a significant 3-mmHg reduction in nighttime SBP that translated into the improvement of the nighttime SBP goal, from 18.0% at baseline to 27.0% at Week 12. Furthermore, a decrease in daytime SBP (2.5 mmHg) and office SBP (7.9 mmHg) led to improved prevalence of SBP control (Fig. 1). Notably, the study also evidenced the amelioration of circadian BP rhythm, with restoration of the BP dipping profile in half of the cohort.

A previous meta-analysis has shown that in diabetic patients without CKD, the use of SGLT2i is associated with an average decrease in nighttime and daytime SBP by 2.6 mmHg and 4.4 mmHg, respectively [6]. The findings of our analysis therefore extend the benefits of SGLT2i on ABP profile to the patients with renal impairment. It is important to note that the nighttime SBP reduction observed in our cohort of DKD patients is comparable to that reported in diabetic patients without renal impairment, while the effect on daytime SBP was less pronounced in our cohort.

The significant reduction in nighttime SBP observed in our study may be attributed to the enhanced natriuretic effect associated with SGLT2i, which may improve sodium and volume overload [2, 18, 19]. These findings align with a previous meta-analysis in diabetes-no CKD, which indicated that the nighttime SBP-lowering effect of SGLT2i is comparable to that of low-dose hydrochlorothiazide, suggesting a diuretic-like effect of these drugs on BP [7]. Consequently, it is conceivable that mitigating sodium overload could impact the circadian BP rhythm and, in particular, nocturnal hypertension, as previously evidenced in sodium-sensitive hypertensive disorders, including CKD [20–22]. The average 2-kg reduction in body weight after 12 weeks of dapagliflozin treatment further supports this hypothesis, though we did not report differences among patients under diuretics or under low salt diet (Fig. 1). Indeed, the natriuretic effect of SGLT2i is likely transitory probably because of over-reabsorption in the distal nephron [23], as expected for all the diuretics acting in the proximal segments of tubules. Furthermore, the absence of a significant correlation between weight loss and nocturnal SBP reduction is not surprising because SGLT2i lower body weight also, by reducing fat mass due to the negative calorie balance [24].

By increasing sodium excretion in the distal tubules, SGLT2i restore tubule–glomerular feedback and, in turn, reduce glomerular hyperfiltration, thus improving “kidney health” [25]. This effect on intraglomerular hemodynamics is the main driver of reduction in albuminuria explaining a substantial part of the positive outcomes on CKD progression [26–28]. In agreement with these trials, we registered a significant effect on albuminuria after the first 12 weeks of dapagliflozin therapy. We reported a greater nighttime SBP reduction in patients with albuminuria >300 mg/day compared with those with lower albuminuria. The explanation for this finding is not readily apparent; we can only speculate that SGLT2i may exert a greater effect on reducing nocturnal hypertension in patients with more pronounced glomerular hyperfiltration who are often characterized by higher albuminuria [28]. This effect on glomerular hyperfiltration may have led to a temporary decrease in GFR during the first 2–4 weeks, which became no longer detectable after 3 months, as testified by the absence of difference in GFR at the end of our study period (*P* = .297).

An interesting finding from our analysis was the improvement of the global BP profile associated with dapagliflozin therapy. We reported an attenuation of the white-coat effect, commonly detected in CKD [15], which was strongly related to the major effect on office SBP (−7 mmHg) as compared with daytime SBP. Of note, the greater decrease in office SBP compared with ABP agrees with data from a meta-analysis of 52 trials involving nearly 10 000 hypertensive patients who were treated with at least one antihypertensive drug [29]. This analysis revealed a difference as high as 6.5 mmHg in the antihypertensive effect when comparing office and ambulatory BP measurements. It is possible that the difference may relate to different factors, that is, the higher levels of office BP vs ABP as well as the white coat effect and the regression to the mean phenomenon that typically affect office BP more.

The improvement in masked and sustained hypertension also has important prognostic implications because both patterns associate with poor outcomes in patients with CKD [8, 9], These findings may be the consequence of a general improvement of hypertensive burden due to SGLT2i, but it may also depend on the higher accuracy in properly defining pressor and circadian profiles due to ABP reassessment [30].

More importantly, the observed effect of SGLT2i on nocturnal hypertension and circadian BP rhythm may contribute to the multiple mechanisms underlying the favorable renal and cardiovascular outcome in DKD. Among patients who completed the study, we registered a BP-lowering effect able to improve the circadian profile in the absence of modifications of anti-hypertensive therapy. Of note, during the 3 months of follow-up, we registered only three cases of AKI due to volume depletion, and four patients with urinary tract infections, confirming the safety profile reported in randomized trials [5].

Finally, despite the short-term observation, we still observed a significant decrease in albuminuria, although to a lesser extent as compared with that reported in the Dapagliflozin and Prevention of Adverse Outcomes in Chronic Kidney Disease (DAPA-CKD) trial [26]. Similar results on albuminuria have been attained in a pooled analysis of randomized controlled trials evaluating the effect of dapagliflozin in patients with type 2 diabetes and stages 3b–4 CKD characterized by lower levels of albuminuria (urine albumin–creatinine ratio 40 mg/g) [31]. This observation is critical when considering that residual proteinuria in DKD, even if low, is predictive of a higher risk of progression to ESKD [32].

The main limitations of this study include its observational nature, the lack of a control group, and the potential impact of regression to the mean on our findings. However, we designed a quasi-experimental study that may offer advantages over experimental designs in terms of being closer to real world clinical practice and cost-effective [33, 34]. Additionally, we adjusted for baseline values of nighttime SBP; this may help to further mitigate the effects of regression to the mean that are already minimal for ABP measures [35]. Furthermore, the seasonal changes in ambulatory BP may act as possible confounder in repeated ABPs in Mediterranean area; however, the greatest effect of SGLT2i was detected in the nighttime component of BP, where the effects of climate are small [36]. Finally, the coexistence of DM and CKD does not prove the presence of true diabetic nephropathy.

In conclusion, in patients with DKD undergoing polytherapy in the setting of nephrology care, we found that starting dapagliflozin associates with a significant improvement in nocturnal hypertension and a higher probability of restoring circadian BP profile, with no significant safety signal. The amelioration of altered BP patterns may act as additional mechanism to be considered in the general picture of SGLT2i-related amelioration of kidney and cardiovascular outcomes.

## Data Availability

The data underlying this article will be shared on reasonable request to the corresponding author.

## Notes

Collaborators are listed in the Acknowledgements.
